# The association between general practitioners’ attitudes towards breast cancer screening and women’s screening participation

**DOI:** 10.1186/1471-2407-12-254

**Published:** 2012-06-18

**Authors:** Line Flytkjær Jensen, Thomas Ostersen Mukai, Berit Andersen, Peter Vedsted

**Affiliations:** 1 The Research Unit for General Practice, Department of Public Health, Aarhus University, Bartholins Allé 2, 8000, Aarhus C, Denmark; 2 Section for General Medical Practice, Department of Public Health, Aarhus University, Bartholins Allé 2, 8000, Aarhus C, Denmark; 3 The Research Centre for Cancer Diagnosis in Primary Care (CaP), Bartholins Allé 2, 8000, Aarhus C, Denmark; 4 Department for Public Health Programs, Randers Regional Hospital, Skovlyvej 1, 8930, Randers NØ, Central Denmark Region, Denmark

**Keywords:** Breast cancer screening, Participation, General practice

## Abstract

**Background:**

Breast cancer screening in Denmark is organised by the health services in the five regions. Although general practitioners (GPs) are not directly involved in the screening process, they are often the first point of contact to the health care system and thus play an important advisory role. No previous studies, in a health care setting like the Danish system, have investigated the association between GPs’ attitudes towards breast cancer screening and women’s participation in the screening programme.

**Methods:**

Data on women’s screening participation was obtained from the regional screening authorities. Data on GPs’ attitudes towards breast cancer screening was taken from a previous survey among GPs in the Central Denmark Region. This study included women aged 50-69 years who were registered with a singlehanded GP who had participated in the survey.

**Results:**

The survey involved 67 singlehanded GPs with a total of 13,288 women on their lists. Five GPs (7%) had a negative attitude towards breast cancer screening. Among registered women, 81% participated in the first screening round. Multivariate analyses revealed that women registered with a GP with a negative attitude towards breast cancer screening were 17% (95% CI: 2-34%) more likely to be non-participants compared with women registered with a GP with a positive attitude towards breast cancer screening.

**Conclusion:**

The GPs' attitudes may influence the participation rate even in a system where GPs are not directly involved in the screening process. However, further studies are needed to investigate this association.

## Background

Breast cancer is the most frequent cancer type and the second most common cancer-related cause of death in Danish women [1,2]. In 2009, the incidence rate was 184 in 100,000 women and the mortality rate 38.4 in 100,000 women. Furthermore, the one- and five-year breast cancer survival rates in Denmark are among the lowest in comparable countries [3,4].

Although the overall effect of breast cancer screening has been discussed [5], breast cancer screening was introduced in Denmark with the aim of increasing breast cancer survival. A high participation rate is crucial to achieve the maximum effect of organised breast cancer screening [6]. Many studies have therefore explored factors affecting participation, including socio-demographic factors, psychosocial factors and practical factors, such as distance to screening site [7-9]. The role of the general practitioner (GP) has also been studied in relation to screening uptake. Both GP age and gender have been shown to be associated with screening uptake [10-12]. Furthermore, most studies have found that women are more likely to participate if encouraged by their GP [13-19], and one review even concluded that mammography recommendation by primary care providers was one of the most important factors for participation [20]. Most of these studies were conducted in the USA [15-17,19], but their results are also supported by studies from other countries, e.g. Sweden [18], Canada [14] and Australia [13].

In the Danish health care system, there is a strong tradition of the GP acting as a gatekeeper and thus as ‘physician of first contact’. On average, women aged 50–70 years consult their GP 6–10 times annually [21]. Despite the close relationship and continuity of care between patients and GPs, breast cancer screening in Denmark is organised by the secondary health care sector, and the women are invited to attend by a central booking service which also handles re-bookings and cancellations. The GP is not responsible for recruiting the women for breast cancer screening and it is unknown whether the GP’s attitude towards screening affects women’s participation. Hence, this study aims to investigate the association between the GP’s attitude towards breast cancer screening and the women’s participation in the programme.

## Methods

### Setting

The setting of this population-based study was the Central Denmark Region (1.2 million inhabitants). The first breast cancer screening round was conducted from February 2008 to December 2009. During this period, all women aged 50–69 years (n = 149,234) were invited to participate by the Department for Public Health Programs, Central Denmark Region. The invitation letters were sent out so that women registered with the same GP were invited at the same time. Women were offered a pre-booked mammogram appointment at one of the region’s five screening sites. The women could change the appointment and site, and they could also decline participation. Non-participants received no reminders.

### Data and variables

#### Data on women’s screening participation

From the Department for Public Health Programs, Central Denmark Region, data was retrieved on all women invited for the first screening round. The data contained information on scheduled screening date, participation and the practice registration number of the GP with whom the woman was registered. The dependent variable in this study was women’s participation in the first screening round in the Central Denmark Region, operationalised as ‘participants’ and ‘non-participants’. Non-participants were further divided into ‘active non-participants’, defined as women who actively called and declined to participate, and ‘passive non-participants’, defined as women who just did not show up for their screening appointment.

Data on women’s socio-demography was used to adjust for factors known to influence screening participation. These data was obtained from ‘The Danish Integrated Database for Labour Market Research’ (IDA) run by Statistics Denmark [22]. The following variables were used: ‘OECD-adjusted household income’ [23] divided into tertiles and rounded off to the nearest 100 euros, ‘ethnicity’ coded as Danish, immigrants from western countries, and immigrants from non-western countries, and ‘marital status’ coded as married, cohabiting, or single. Based on information from the Department for Public Health Programs, women’s ages were calculated on the scheduled screening date. Distance to screening site in kilometres, based on the shortest route, was also used as a variable and was calculated according to the Danish road network using ArcGIS Network Analyst (version 10.0) [24]*.* Geographical coordinates obtained from the Centralised Civil Register were used to locate each woman’s residence.

#### Data on GPs’ attitudes towards breast cancer screening

Data on the GPs’ age, gender and attitude towards breast cancer screening was obtained from a questionnaire used in another study aiming to measure GPs’ involvement and need for information about breast cancer screening in primary care. Part of this questionnaire concerned the GPs’ attitudes towards breast cancer screening. The GPs were asked if they had a positive, negative, or indecisive attitude of breast cancer screening. Information on practice type (singlehanded or partnership) was obtained from the Danish National Board of Health. Nearly all (98%) Danish citizens are listed with a GP practice of which approximately 60% are singlehanded practices. The GPs in partnership practices (66% of all GPs) most often work in practices with two to four GPs. In most partnership practices, GPs formally share the patient list but in reality have their own patients. As we thus could not link a specific patient to an individual GP in a partnership practices, only women registered with a singlehanded practice were included.

#### Study period and data collection

The study was conducted during the latter months of the first screening round. Between February and October 2009, the GP questionnaire was sent to 330 singlehanded and partnership GPs (87 singlehanded GPs and 243 partnership GPs). GP questionnaires were mainly sent out during the period of time where the women on their lists had their bookings for screening. A reminder, including a new questionnaire, was sent to non-responding GPs after two weeks. 80% of the women were screened before their GP filled in the questionnaire, but 50% of these women had a screening appointment within a maximum of 40 days before the GPs answered the questionnaire (90% tertile =117 days). GP questionnaires were scanned and verified using Cardiff Teleform software. Data on women’s characteristics (participation and socio-demographic and economic position) was linked using each woman’s unique personal identification number allocated to all Danish citizens. Finally, data on GP attitude, age and gender was linked to each woman using the GP's practice registration number.

According to Danish Legislation and the Central Denmark Region Committees on Biomedical Research Ethics (j.no.: 181/2011) the study should not have a formal ethical approval, as it was based on registry data. The project was approved by the Danish Data Protection Agency (j.no.: 2009-41-3471 and j.no.: 1-16-02-31-11).

### Analyses

Generalised linear models with log link and the Bernoulli family regression model [25,26] were used to estimate the association between GPs' attitudes towards breast cancer screening and women’s participation rates. Three multivariate regression analyses were made. The first model adjusted for GP age and gender, the second model for women’s characteristics, and the third model further adjusted for women's distance to screening site. Using the same models, sub-analyses were conducted to investigate whether there was an association between the GPs’ attitudes and the women’s active or passive non-participation. Prevalence ratios (PRs) with 95% confidence intervals (95% CI) were used as an association measure using robust variance estimates to adjust for clustering of patients within general practices in both unadjusted and adjusted models [27]. Statistical analyses were conducted using STATA ver. 11.

## Results

### GP characteristics

The response rate to the GP questionnaire was 77% (n = 67) among singlehanded GPs who had a total of 13,288 women registered on their lists during the study period from February 2009 to October 2009. Mean GP age was 56 years, and 79% were male. Fifty-three of the singlehanded GPs (79%, 95% CI: 69-89%) had a positive attitude towards screening, five were negative (7%, 95% CI: 1-14%), and nine were indecisive (13%, 95% CI: 5-22%).

### Invited women’s characteristics

The characteristics of the women registered with the 67 GPs are shown in Table 1. Compared with the participants, more non-participants had a GP with a negative (6.8 vs. 8.4%) or an indecisive attitude (13.2 vs. 16.2%) towards screening (p < 0.001).

**Table 1:** Distribution of participants (n = 10,773) and non-participants (n = 2,515) in a breast cancer screening programme in relation to GPs’ and women’s characteristics

	**Participants**		**Non-participants**		***P-*value**
	**N**	**% (column)**	**N**	**% (column)**	
**GP’s attitude**					
Positive	8,615	(80.0)	1,897	(75.4)	<0.001
Negative	732	(6.8)	212	(8.4)	
Indecisive	1,426	(13.2)	406	(16.2)	
**GP’s gender**					
Male	8,479	(78.7)	2,010	(79.9)	0.179
Female	2,294	(21.3)	505	(20.1)	
**Women’s age**					
50-54	2,942	(27.3)	600	(23.9)	<0.001
55-59	2,801	(26.0)	608	(24.2)	
60-64	2,786	(25.9)	634	(25.2)	
> = 65	2,244	(20.8)	673	(26.7)	
**Household income**					
Low	3,193	(29.7)	1,233	(49.2)	<0.001
Middle	3,711	(34.5)	715	(28.5)	
High	3,866	(35.9)	560	(22.3)	
**Marital status**					
Married	7,557	(70.2)	1,309	(52.2)	<0.001
Cohabiting	756	(7.0)	192	(7.7)	
Single	2,450	(22.8)	1,006	(40.1)	
**Ethnicity**					
Danish	10,449	(97.0)	2,322	(92.5)	<0.001
Western immigrants	189	(1.8)	79	(3.2)	
Non-western immigrants	131	(1.22)	109	(4.3)	
**Distance to screening site**					
0-20 km	5,888	(57.2)	1,281	(53.1)	<0.001
>20-40 km	2,555	(24.8)	575	(23.8)	
>40-60 km	1,508	(14.6)	422	(17.5)	
>60 km	347	(3.4)	136	(5.6)	

Numbers vary due to missing data.

### Association between GPs' attitudes and women’s participation

Women were statistically significantly more likely to be non-participants (Table 2) if their GP had a negative attitude towards screening than women registered with a GP with a positive attitude. This association was not altered when controlling for GP age and gender (Model 1). Also when adjusted for women’s socio-demographic characteristics (Model 2), the estimate was stable and remained statistically significant. Adjusting for the women’s distance to the screening site (Model 3) reduced the association, which was still statistically significant, however, and indicated a 17% (95% CI: 1.02-1.34) higher likelihood of non-participation among women registered with GPs with a negative attitude. The association between having a GP with an indecisive attitude towards screening and women’s participation was not statistically significant in any of the models (Table 2).

**Table 2:** Association (prevalence ratio (PR)) between GPs’ attitudes towards screening and women’s non-participation in a breast cancer screening programme

	**Crude**	**Model 1**	**Model 2**	**Model 3**
		GP’s Characteristics	Women’s characteristics	Model 2 + distance
**Attitude**				
Positive	1 (ref)	1 (ref)	1 (ref)	1 (ref)
Negative	1.24 (1.06-1.47)**	1.25 (1.07-1.46)**	1.23 (1.07-1.41)**	1.17 (1.02-1.34)*
Indecisive	1.23 (0.99-1.53)	1.22 (0.98-1.52)	1.17 (0.97-1.40)	1.10 (0.96-1.26)
**GP’s gender**				
Male	1 (ref)	1 (ref)	-	-
Female	1.06 (0.89-1.26)	1.05 (0.88-1.26)		
**GP’s age**^**1**^	1.00 (0.99-1.01)	1.00 (0.99-1.01)	-	-
**Women’s age**				
50-54	1 (ref)	-	1 (ref)	1 (ref)
55-59	1.05 (0.94-1.18)		1.06 (0.95-1.18)	1.07 (0.96-1.19)
60-64	1.09 (0.97-1.23)		1.04 (0.93-1.17)	1.04 (0.93-1.17)
65+	1.36 (1.20-1.54)**		1.11 (0.98-1.26)	1.12 (1.00-1.25)
**Income**^**2**^				
Low	1 (ref)	-	1 (ref)	1 (ref)
Middle	0.58 (0.53-0.64)**		0.66 (0.60-0.72)**	0.67 (0.61-0.73)**
High	0.45 (0.41-0.51)**		0.58 (0.51-0.65)**	0.58 (0.52-0.65)**
**Marital status**				
Married	1 (ref)	-	1 (ref)	1 (ref)
Cohabiting	1.37 (1.21-1.55)**		1.41 (1.24-1.60)**	1.40 (1.24-1.59)**
Single	1.97 (1.82-2.13)**		1.61 (1.49-1.75)**	1.62 (1.49-1.76)**
**Ethnicity**				
Danish	1 (ref)	-	1 (ref)	1 (ref)
Western immigrants	1.62 (1.36-1.93)**		1.46 (1.23-1.74)**	1.43 (1.19-1.71)**
Non-western immigrants	2.50 (2.16-2.89)**		1.91 (1.63-2.23)**	1.99 (1.73-2.28)**
**Distance to screening site**				
0-20 km.	1 (ref)	-	-	1 (ref)
>20-40 km.	1.03 (0.89-1.19)			1.05 (0.92-1.19)
>40-60 km.	1.22 (1.02-1.47)*			1.19 (1.03-1.38)*
>60 km.	1.58 (1.33-1.87)**			1.52 (1.32-1.76)**

^1^ Numeric

^2^ Divided in tertiles based on OECD-adjusted household income the year prior to screening (in euros).

*p < 0.05.

**p < 0.01.

Model 1 – adjusted for GP age and GP gender.

Model 2 – adjusted for women’s age, OECD-adjusted household income, marital status, and ethnicity.

Model 3 – adjusted for women’s age, OECD-adjusted household income, marital status, ethnicity, and distance to screening site.

Sub-analyses of the non-participant group revealed no statistically significant association between women’s active or passive non-participation and the GPs' attitudes towards breast cancer screening (data not shown).

## Discussion

Women registered with a GP with a negative attitude towards breast cancer screening were more likely to be non-participants compared with women registered with a positive GP. Controlling for women’s socio-demographic characteristics and for distance to screening site reduced the association, but it remained statistically significant.

Although women were invited based on the GP practice with which they were registered, these results were still somewhat surprising given the fact that bookings, investigations and follow-up of the screening programme did not involve general practice. Although the confidence interval was fairly close to 1.0 in the multivariate analyses, fairly similar prevalence ratios were observed across different statistical models. One explanation could be that the women feel more comfortable consulting their GP than the booking service for advice if they are uncertain whether or not to take part in the screening programme due to the central role of the primary health care system in Denmark. The attitude of the GP is therefore more likely to influence the women’s choice of participation.

Sub-analyses showed no statistical difference between active and passive non-participants and their GPs’ attitudes towards screening. Hence, no association was observed between GPs’ attitudes and whether or not the women were more likely to call and cancel their appointment (active non-participation) or not to show up (passive non-participation). It should be noted, however, that since these analyses were conducted only on non-participants (n = 2,515), some of the groups were rather small.

The results of this study are consistent with findings from studies in other countries indicating that the GPs' influence on screening participation seems to be universal despite different ways of organising the programme worldwide [13,16,18]. However, the results of this study are not as clear as those seen in the USA where advice from health care professionals is regarded as one of the most important determinants for screening participation [20]. Results similar to those of the present study have been seen in Sweden [18]. This supports the conclusion of the Swedish study that the GP has an influence on participation in population-based outreach mammography screening programmes.

It should be noted that we do not know to what extent the women consulted their GP for advice on screening participation. Also, on the basis of this observational study, it is not possible to make causal inferences as the actual interaction between the GPs and the women remains unknown. One study, however, has indicated that GPs with a positive attitude are more likely to recommend screening than GPs with a negative attitude [10].

The strength of this study is the large population-based design where information about screening participation and the women’s characteristics were obtained from valid and complete registers. This minimises the risk of selection and information bias. An additional strength is that data on screening participation was collected during the first screening round in the region, which makes the population ideal for studying the effect of GPs’ attitudes since no women were excluded on the grounds that they had previously actively chosen not to take part in the programme. The response rate among the GPs was high although only 67 GPs participated, of which only five were negative towards screening. Selection bias may be present since it cannot be ruled out that GPs with special characteristics were more likely to respond or not respond to the questionnaire, e.g. it is plausible that negative GPs were more likely to be non-responders. In addition, women registered with singlehanded GPs may represent women with special characteristics. This is indicated by a somewhat higher participation rate (81.1%) among the population included in our study compared with the entire population of women invited to the first screening round (78.7% excluding the study population – data not shown).

The method used to measure the GPs’ attitudes may be a limitation of the study. Data on the GPs’ attitudes were collected in another study which included only a single categorical question about the GPs’ attitudes. It might have been advantageous to use a more comprehensive measure to assess the GPs’ attitude. Furthermore, due to the data collection method used in this study, 80% of the women were offered screening before their GP answered the questionnaire assessing their attitudes. It cannot be excluded that the attitude of the GP may have been affected and even ultimately changed as a consequence of the screening round, which could lead to information bias.

Only singlehanded GPs were included in this study. If we had included women registered with partnership GPs, the number of included GPs would have been larger and the statistical accuracy higher. However, including partnership GPs could seriously bias the study, as we could not link a GP to the women in partnership practices and therefore also do not know the attitude of the particular GP whom a woman sees. By only including singlehanded GPs we knew the attitude of the GP the woman had seen if she had sought advice from her GP during this period. Future studies should be designed to measure the association between the attitude towards breast cancer screening among partnership practices and women’s screening participation.

## Conclusion

The GPs’ attitudes towards breast cancer screening were statistically significantly associated with women’s participation in breast cancer screening when adjusted for socio-demographic differences and distance to screening site. However, the observed association was not as strong as observed in other studies. Our findings should be seen in the light of the limitations of this study. Studies including a larger sample of GPs, retrospectively measuring GPs’ attitudes to screening participation and using a more comprehensive measure to assess the GPs’ attitudes are needed to further clarify the specific association between GPs’ attitudes and women’s participation.

## Competing interests

The authors declare that they have no competing interests.

## Authors’ contributions

LFJ, TOM, BA, and PV all conceived the study, participated in its design and helped to draft the manuscript. TOM conducted the GP survey. All authors read and approved the final manuscript.

## Pre-publication history

The pre-publication history for this paper can be accessed here:

http://www.biomedcentral.com/1471-2407/12/254/prepub
